# Artificial Intelligence in Outpatient Primary Care: A Scoping Review on Applications, Challenges, and Future Directions

**DOI:** 10.1007/s11606-025-09938-0

**Published:** 2025-10-28

**Authors:** Stacy Iannone, Amarpreet Kaur, Kevin B. Johnson

**Affiliations:** 1 Division of Biomedical Informatics, Department of Biostatistics, Epidemiology, and Informatics, Perelman School of Medicine at the University of Pennsylvania, Philadelphia, PA USA; 2 Clinical Futures Lab, Department of Pediatrics, Children’s Hospital of Philadelphia, Philadelphia, PA USA; 3 Division of Biomedical Informatics, Department of Biostatistics, Epidemiology, and Informatics, Perelman School of Medicine at the University of Pennsylvania, Philadelphia, USA; 4 Department of Pediatrics, Children’s Hospital of Philadelphia, Philadelphia, PA USA

**Keywords:** scoping review, artificial intelligence, clinical decision-making, outpatient care, health information systems, patient care management, primary care

## Abstract

**Background:**

Artificial intelligence (AI) has significant potential to impact clinical decision-making and improve patient outcomes in outpatient primary care. However, despite rapid advancements, the extent of AI implementation in outpatient primary care remains unclear. This scoping review explores how AI functions, undergoes trials, or integrates into non-urgent outpatient primary care settings.

**Methods:**

This scoping review was conducted in accordance with the Joanna Briggs Institute methodology and reported following the PRISMA-ScR (Preferred Reporting Items for Systematic Reviews and Meta-Analyses Extension for Scoping Reviews) guidelines. We searched MEDLINE, CINAHL, Scopus, and clinicaltrials.gov databases. Eligible studies were peer-reviewed articles published in English between January 2019 and November 22, 2024, examining AI applications in primary care settings with a direct focus on patient care. Studies were excluded if they were not in English, did not address primary care workflows, or if the full text was unavailable. We added clinicaltrials.gov to uncover active protocols that suggested wider potential adoption. We used thematic analysis to synthesize findings related to AI application domains, research stage, and status of implementation.

**Results:**

We screened 3203 manuscripts and found 61 that met the eligibility criteria. Most studies (*n* = 26; 43%) focused on model development, while eight reported clinical trial results. AI applications included provider support (*n* = 5; 8%) and radiological disease diagnosis (*n* = 1; 2%). Most studies examined clinical decision-making, disease diagnosis, and risk prediction, but none addressed provider cognitive support, workflow automation, or risk-adjusted paneling. There were 11 studies of real-world implementations.

**Conclusion:**

Overall, based on this scoping review of peer-reviewed literature, AI in primary care remains in the developmental stage, with minimal real-world use beyond ambient scribing, clinical decision support, and workflow automation.

**Supplementary Information:**

The online version contains supplementary material available at 10.1007/s11606-025-09938-0.

## BACKGROUND

As the National Academy of Medicine noted, integrating advanced artificial intelligence (AI) technologies into healthcare may offer an unprecedented opportunity to transform patient care.^1,2^ The literature contains numerous examples showcasing the promise of AI, including systematic reviews to assess potential roles in clinical care and research.^3–6^ More recently, the release of AI tools powered by large language models (LLMs), such as ChatGPT, has led to a dramatic increase in tools that can augment the creation of medical information for specific purposes, such as drafting patient portal message responses, information summarization, and enabling ambient scribing in outpatient care settings.^7–9^

Despite AI’s demonstrated potential in hospital settings, much less is known about its current applications, opportunities, and challenges in outpatient primary care and ambulatory contexts.^10,11^ As Lin and colleagues have noted, mature AI systems hold promise for transforming primary care delivery through innovations in diagnostic support, clinical decision-making, risk prediction, documentation assistance, data integration, practice management, and offering other forms of cognitive support to primary care providers.^12^ However, it is not yet clear how comprehensively these opportunities have been explored in primary care; nor is it clear where along the pipeline—from early ideation and conceptual work through development, pilot testing, and operational use—most research is concentrated, but the distribution and depth of existing studies remain poorly characterized.

This uncertainty about the penetration of AI in primary care makes conducting a scoping review particularly important. Existing literature should provide an efficient view of the feasibility and reach of AI in primary care, identify how published studies align with theoretical opportunities, and map their placement along the development-to-implementation pipeline.

## OBJECTIVE

This scoping review examines the coverage (breadth of use) and penetration (extent of adoption) of AI technologies in outpatient internal medicine, family medicine, and pediatric primary care settings. Specifically, it aims to summarize the frequency with which studies report AI use and the specific capacities in which it is applied (e.g., diagnostic support, workflow assistance, documentation, communication) based on a framework proposed by Lin and colleagues.^12^

## MATERIALS AND METHODS

We conducted the scoping review in accordance with the Joanna Briggs Institute methodology and reported following the PRISMA-ScR (Preferred Reporting Items for Systematic Reviews and Meta-Analyses Extension for Scoping Reviews) guidelines.^13^ We selected this methodology because it aligns with our objective to catalogue all uses of AI tools in primary care settings.

### Search Strategy and Selection Criteria

We conducted a comprehensive search across PubMed, MEDLINE, Scopus, CINAHL, and for English-language studies published in peer-reviewed journals and research studies between January 01, 2019, and November 22, 2024. We developed our search strategy in collaboration with librarians at the University of Pennsylvania, Perelman School of Medicine, specializing in medical science and engineering. Our search strategy used a combination of key terms, subject headings, and standard abbreviations broadly related to AI, primary care, ambulatory care, and patient care. We added a search of Clinicaltrials.gov to complement published protocols, which represent the leading edge of clinically relevant research that is a harbinger of larger scale deployment of AI. Detailed inclusion and exclusion criteria are outlined in Table 1. Supplemental Appendix 1, Table A, summarizes our final search strategy for each database used.

**Table 1:** Eligibility Criteria Used in the Scoping Review

Category	Inclusion criteria	Exclusion criteria
Document type	Peer-reviewed publications Primary studies Research articles	Physician letters or notes Book or book chapter Reviews (ex: systematic reviews, scoping reviews) Surveys
Population	Ambulatory setting Outpatient setting Primary care Mental healthcare in ambulatory setting Rehabilitative care in ambulatory setting EHR analysis within ambulatory clinical care (using EHR with AI)	Inpatient setting Emergency department Nursing care facilities End-of-life care Palliative care Specialty care or condition (ex: COPD, neurology) EHR analysis external to ambulatory care settings Focus groups
Subject area	Medicine Clinical informatics/engineering Neuroscience Psychology Dentistry	Non-healthcare related fields (ex: environmental sciences, agricultural sciences, social sciences) Nursing Pharmacology and pharmaceutics Veterinary Health education Healthcare management, finance or policies
Intervention/exposure	Artificial intelligence Deep learning Machine learning Neural networks	Any technology without explicit mentioning of AI/ML, or AI/ML designed for other primary care stakeholders (e.g., billing, education, research professionals)
Study design	Peer-reviewed research, protocols, clinical trials	Editorials, reviews, abstracts

### Study Selection

Our review team (SLI, AK, KBJ) established inclusion and exclusion parameters prior to developing the search strategy and iteratively refined them to focus on the most relevant studies. We included peer-reviewed studies examining the use of AI/ML by healthcare providers in primary care settings. We focused on empirical studies that evaluated or tested the use of AI technologies in outpatient primary care or ambulatory care settings. We excluded studies that only described algorithms without application to primary care, those limited to inpatient settings, and non-peer-reviewed opinion or commentary pieces.

We used Covidence, a web-based tool designed to streamline systematic reviews,^14^ to aid in screening and selecting unique articles based on the specified criteria. All three authors conducted the screening and selection process, ensuring they followed the PRISMA-ScR guidelines’ two-step approach. The team first screened articles based on titles and abstracts. To ensure consistency and to refine the inclusion and exclusion criteria, we conducted two trial runs, starting with 50 articles from PubMed, to align the reviewers’ understanding of the literature. Following consensus on the initial sample from PubMed, the team screened another 50 articles from Scopus to check for reduced inter-reviewer differences, which successfully diminished. Once alignment was achieved, the two reviewers proceeded to screen the remaining articles by title and abstract.

We excluded studies focused on inpatient care, emergency departments, nursing facilities, end-of-life or palliative care, and specialty conditions unlikely to be managed in primary care (e.g., neurology). Additionally, studies involved pharmacology, nursing, veterinary medicine, and non-healthcare sectors—e.g., environmental, agricultural, and social sciences. Data sources such as physician letters, book chapters, reviews, surveys, and focus groups were also excluded.

After removing duplicates, the three reviewers (SLI, AK, KBJ) independently screened article titles and abstracts for relevance using Covidence. Two authors evaluated each abstract reviewed against the eligibility criteria, with a third reviewer serving as a tiebreaker in cases of disagreement. We included full-text articles in the final analysis if the two initial reviewers reached a concordant decision or, in cases of discordance, after all three reviewers reexamined the article and resolved the disagreement by consensus.

In the second step, two reviewers conducted a full-text review of the articles identified during the initial screening, carefully assessing all articles by applying the established inclusion and exclusion criteria to determine final eligibility.

### Data Extraction and Synthesis

A standardized data chart was developed to ensure consistency in data extraction across all included studies.

- Article citation details (authors, title, year)
- Country and targeted health organization (when applicable)
- Focus areas of AI application (not limited to “innovation”):

We identified these domains a priori based on relevance to outpatient primary care workflows and patient care processes, as defined by Lin and colleagues 12.

- Primary care provider cognitive support
- Practice management
- Clinical decision-making
- Disease diagnosis
- Chart review/documentation
- Wearable integration
- Risk-adjusted paneling
- Medical advice
- Population health
- Risk prediction
- General discussion

- Implementation phase:

We categorized each paper’s stage of implementation, as defined by Kanbar.^15^ We added “protocol” as one way that trials may appear in the published literature before they are completed.

- Development
- Validation
- (Protocol)
- Trial
- Clinical use (descriptive or observational)

## RESULTS

**Figure 1:**
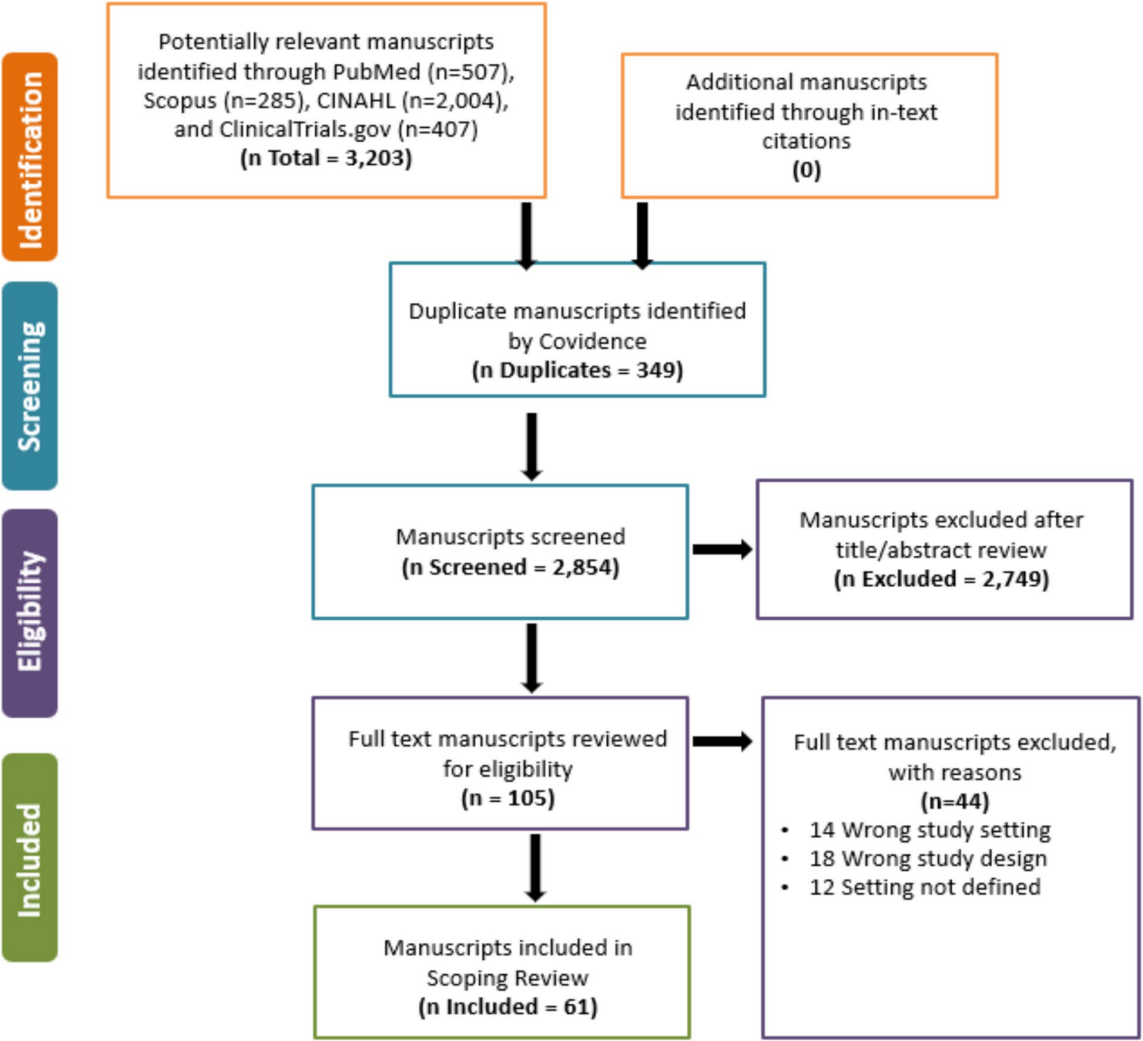
PRISMA flow diagram for a scoping review of eligible studies, modified to include the use of clinicaltrials.gov to identify work being tested through implementation.

Figure 1 presents the PRISMA-ScR screening and selection process. We identified 3,203 potentially relevant manuscripts through our search: PubMed (*n* = 507), Scopus (*n* = 285), CINAHL (*n* = 2004), and Clinicaltrials.gov (*n* = 407). We did not find any additional references meeting our inclusion criteria through in-text citations. After we eliminated duplicates, we screened 2,854 titles and abstracts and reviewed 105 in full text. We resolved all disagreements about article inclusion by consensus. Sixty-one studies met all criteria, and we included them in the final analysis.

A complete list of included studies is provided in Supplemental Appendix A.

### Study Characteristics

Appendix 1, Supplemental Figure A provides an overview of the journals in which the included articles were published. The articles were published in various journals, including specialty journals focused on oncology, orthopedics, endocrinology, cardiology, and informatics.

Appendix 1, Supplemental Table D summarizes the key characteristics of the included publications, using a framework outlining the potential impact of AI on primary care.^12^ Manuscripts originated from investigators in the USA (*n* = 35), Europe (*n* = 8), and other countries (*n* = 9); among these, 19% focused on family medicine, 35% on internal medicine, and 8% on pediatrics. Studies were conducted in various settings, including primary care within health systems, private practices, and other outpatient environments.

### Coverage of AI in Primary Care

We conducted an analysis of the full text for each study and assigned each study to one primary goal of the AI model being discussed. Of the 61 studies included, 15 (25%) focused on *disease diagnosis*, and 14 (23%) on *risk prediction*, making these the two most common areas of investigation. *Clinical decision-making* was the aim of 9 (15%) studies, while 5 (8%) targeted *PCP cognitive support*. Smaller proportions addressed *medical advice* (4, 7%), *population health* (3, 5%), *wearable integration* (3, 5%), and *chart review–documentation* (2, 3%). Only 1 (2%) study addressed *practice management*, and no studies were primarily focused on *risk-adjusted paneling* or *general* applications. The distribution of these themes is summarized in Table 2.

#### Theme 1. Disease Diagnosis and Disease Risk Prediction

Many studies examined how AI supports diagnosis and disease risk prediction in primary care. Researchers applied tools to detect cardiovascular disease,^16–20^ diabetes,^21–25^ dermatopathology,^26–28^ cognitive impairment,^29,30^ lung cancer risk,^31^ and mental health conditions.^32–34^ Other areas of focus included rheumatology,^35,36^ fracture detection,^37^ respiratory disease,^3,38–41^ and disease recovery prediction,^42^ end-of-life care.^43^ In pediatrics, studies focused on the prediction of child maltreatment,^44^ otitis media severity,^45^ and respiratory virus disease prediction.^46^ Most studies used retrospective EHR data, while several integrated wearable or patient-reported data.^23,43^ These studies reported improvements in diagnostic accuracy compared to standard care or clinician-only assessments. However, methods varied widely, and very few linked predictions directly to patient outcomes such as reduced hospitalizations or better quality of life.

#### Theme 2. Clinical Decision-Making and Cognitive Support

A smaller set of studies tested AI as a cognitive aid for primary care providers. Studies examined decision support for prescribing and guideline adherence,^47^ personalized treatment planning,^48–55^ mental health crises,^32,56,57^ and documentation automation.^58–65^ Most of these studies were pilot trials or simulations, and they rarely evaluated downstream effects on patient outcomes.

Several recent studies evaluated AI tools that reduce clinician burden and streamline practice workflows. Researchers tested applications such as automated chart review,^66^ natural language processing for documentation, and AI-powered triage for patient messages.^67^ Most reported improved efficiency, but few studies assessed whether efficiency gains allow providers to spend more time with patients or improve patient satisfaction.

**Table 2:** Breadth and Penetration of AI in Primary Care

Category	Ideation	Development	Validation	Protocol	Trial	Use	**Total**
PCP cognitive support	0	0	0	0	0	5	**5**
Practice management	0	1	0	0	0	0	**1**
Clinical decision-making	1	5	2	0	1	0	**9**
Disease diagnosis	0	7	5	0	2	1	**15**
Chart review–documentation	0	0	2	0	0	0	**2**
Wearable integration	0	0	1	1	1	0	**3**
Risk-adjusted paneling	0	0	0	0	0	0	**0**
Medical advice	0	1	0	2	1	0	**4**
Population health	0	1	2	0	0	0	**3**
Risk prediction	0	11	3	0	0	0	**14**
General	5	0	0	0	0	0	**0**
**Total**	**6**	**26**	**15**	**3**	**5**	**6**	**61**

The bold indicates the total

### Extent of Adoption

**Figure 2:**
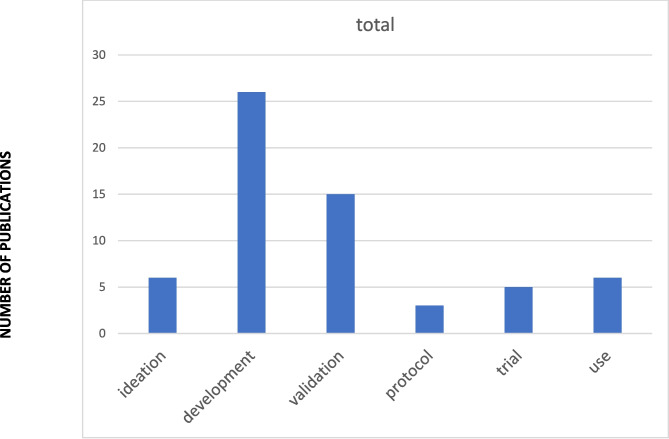
Extent of adoption of AI tools in primary care.

We conducted an analysis of the full text for each study and assigned each study to one of six levels of use. Of the 61 studies included, *Ideation* (conceptual or early exploratory studies) accounted for **6 (10%)** studies,^51,58,67,68^**26 (43%)** were in the *development* (model creation/refinement) stage,^17,21,25,29–31,33,34,36,38–40,44–49,52,53,69–75^and **15 (25%)** were categorized as *validation* (controlled setting testing),^18,19,22,26–28,35,40,42,43,54,57,62,63,66^ emphasizing evaluation of model performance in controlled or retrospective contexts.^18,19,22,26–28,35,42,43,54,57,62,63,66,76^**Three studies (5%)** were identified as *protocols* (registered trials),^55^ while **5 (8%)** reached the *trial* stage, reporting prospective testing in practice environments. These articles focused on tools in cardiology screening,^16^ suicide prevention,^56^ diabetes self-management education,^50^ diabetic retinopathy screening,^24^ automated insulin delivery,^23^ emotional distress counseling,^32^ fracture detection,^37^ and hypertension self-care.^55^ Finally, **6 (10%)** were classified as *use*, describing AI applications implemented and undergoing evaluation in active clinical workflows. Five of these studies investigated the use of generative AI systems in clinical care,^59–61,64,65^ and one publication described the use of an AI-based diagnostic system for COVID-19 pneumonia.^41^ Figure 2 summarizes the extent of adoption for the papers included in this review.

## DISCUSSION

This scoping review of AI applications in primary care revealed several challenges, highlighting both AI’s transformative potential and the complexities associated with integration into routine practice. Our results showed that most studies remain confined to model development, with limited movement toward clinical implementation. We also found that many efforts target the most complex areas of primary care, where integration challenges are inherently higher. Studies often occurred in narrow contexts, such as single sites or small pilot projects, further underscoring that AI’s integration into real-world practice is still in its early stages.

The variability in study quality and the heterogeneity of AI applications complicate interpretation further. Some studies reported efficiency gains or diagnostic improvements, while others raised concerns about workflow disruption or equity implications. Few large-scale planned or ongoing trials exist, which suggests a delay in equitable and widespread deployment. These findings align with broader concerns raised in prior literature, including Kueper and colleagues (2020), about the slow pace of AI integration into primary care.

We included ideation papers in this review because they provided emerging perspectives, theoretical frameworks, and proposed innovations not yet tested in practice. Our results show that these conceptual works contribute to shaping the direction of AI research and highlight anticipated roles for AI in primary care, considering both empirical and ideation studies allowed us to present a comprehensive understanding of AI’s trajectory, while also revealing systemic barriers to translation.

Several systemic issues emerged from the studies reviewed, including challenges in securing NIH funding for non-disease-specific projects, limited willingness among busy practices to test unproven technologies, and the rapid deployment of commercial AI tools without academic validation. Taken together, our findings illustrate not just the promise of AI in primary care but also the pressing need to bridge gaps between development and implementation, and to align research agendas with the realities of frontline practice. A multipronged approach is needed to accelerate the responsible integration of AI into primary care. First, federal and state governments can play a pivotal role by funding large-scale clinical trials to assess AI applications’ safety, efficacy, and fairness. These trials could be led by learning health systems, as proposed by Johnson and colleagues,^77^ or be sponsored by networks of care providers and professional societies, similar to initiatives led by the Pediatric Research in Outpatient Settings Network.^78^

Second, as LLMs such as ChatGPT become more prevalent, concerns around patient mistrust and the risk of AI models generating inaccurate outputs (hallucinations) must be addressed. Establishing rigorous standards for data security, privacy, and transparent reporting of LLM use is critical. Some communities have already proposed frameworks for responsible AI deployment, emphasizing accountability and transparent reporting of AI use in clinical settings.^2,79,80^

Third, AI developers often face challenges accessing primary care sites and data, hindering innovation and real-world testing. Strong partnerships between these AI developers and healthcare delivery experts will be essential. Collaborative efforts between healthcare providers, academic institutions, and technology companies could help bridge this gap while ensuring patient privacy and ethical data use. By fostering these interdisciplinary collaborations, ensuring equitable access to AI resources, and shifting focus toward large-scale implementation, AI can be effectively integrated into primary care, ultimately improving patient outcomes and healthcare delivery that aims to benefit all patients and providers.

### Limitations

This review has several limitations stemming from its reliance on published literature, inherently excluding ongoing or unpublished studies. Consequently, it may provide an incomplete picture of the recent advancements or emerging trends in the field. Additional constraints include the following:

- **Language restriction**: Only studies published in English were included, potentially overlooking relevant findings published in other languages.
- **Lack of unified definitions**: The absence of widely accepted standardized definitions for artificial intelligence (AI), machine learning (ML), and primary care introduces variability in study selection and interpretation.
- **Restricted access**: The review did not incorporate insights from proprietary research, industry driven R&D efforts, or commercial developments, which may significantly influence AI advancements in primary care.
- **Limited scope of literature queries**: Key repositories such as medRxiv, arXiv, and computer science conference proceedings were not queried. While these sources are unlikely to yield translational or clinical trial publications, their exclusion may still have omitted emerging technical innovations.

Moreover, the heterogeneous nature of AI applications across studies adds a layer of complexity. Variations in methodologies, study objectives, and evaluation criteria hinder the comparability of results and may affect the reliability and generalizability of this review’s findings. These disparities limit the ability to generalize conclusions across diverse healthcare contexts, thereby highlighting the need for caution in interpreting the review’s outcomes. One approach that could be done to alert the community to advances in this area is to leverage a period survey strategy focusing on the penetration of AI/ML tools in the primary care ecosystem. A similar strategy has begun to assess cognitive burden with some success.^81^

### Future Directions

Based on this review, future research must prioritize several key areas to advance the responsible integration of AI into primary care. A significant emphasis should be placed on conducting large-scale validation studies, publishing protocols, and disseminating successful AI-enabled tools and approaches to bridge the current gaps between development and clinical adoption. Researchers and information technology (IT) professionals play a vital role as they must address integration challenges to support pragmatic effectiveness trials, particularly those evaluating AI integration within real-world patient care settings. These integration challenges include (A) technical barriers, such as ensuring compatibility with current electronic health records (EHRs) and maintaining robust data security and privacy concerns, and (B) logistical and organizational concerns, including workflow adjustments, clinical staff training, and alignment with existing healthcare processes to promote seamless AI adoption.

Finally, researchers must explore the impact of AI on patient outcomes across diverse populations and healthcare settings. While AI technologies hold immense potential to transform healthcare by providing personalized and efficient care, their effects may vary significantly depending on patient demographics, underlying health conditions, and clinical environments.^82–85^ To ensure equitable patient access and benefits, researchers must investigate how AI impacts different groups, including various age ranges, socioeconomic backgrounds, and geographic locations. Future research prioritized by validation, integration, and inclusivity can drive meaningful advancements in healthcare delivery and patient outcomes, ensuring that AI-driven innovations are practical and broadly beneficial to the primary care ecosystem.

## CONCLUSION

This scoping review of 61 studies highlights the immaturity of AI and ML in primary care, with the predominant state of the research still focusing on development and validation rather than real-world implementation. To accelerate the maturation, testing, and development of AI and ML in primary care, clinical trials must be prioritized, coupled with the advancements of LLMs and the adoption of AI by commercial EHR vendors. These efforts will be critical in bridging the gap between AI innovations and their widespread adoption in clinical practices across diverse healthcare settings.

## Supplementary Information

Below is the link to the electronic supplementary material.

Supplementary Material 1 (DOCX 27.5 KB)

## Data Availability

None.
