# Supplementary material for: Artificial Intelligence in Outpatient Primary Care: A Scoping Review on Applications, Challenges, and Future Directions
Source: J Gen Intern Med. 2025 Oct 28;41(2):364–73. doi: 10.1007/s11606-025-09938-0 (PMC12894555; doi:10.1007/s11606-025-09938-0)
Supplement: Supplementary file 1 — Supplementary Material 1 (DOCX 27.5 KB) [file 11606_2025_9938_MOESM1_ESM.docx]

*Supplemental Table A: Summary of search terms and queries used in each academic literature database*

| Database | Concept | Search String | Operator |
| --- | --- | --- | --- |
| PubMed | Artificial Intelligence/ machine learning | ("Artificial Intelligence"[MeSH Terms] OR "AI"[Title/Abstract] OR "machine learning"[Title/Abstract] OR "deep learning"[Title/Abstract] OR "neural networks"[Title/Abstract] OR ambient[Title/Abstract]) | AND |
|  | primary/ ambulatory care | ("Outpatient Clinics, Hospital"[MeSH Terms] OR "outpatient"[Title/Abstract] OR "outpatient medicine"[Title/Abstract] OR "primary care"[Title/Abstract] OR "family medicine"[Title/Abstract] OR "ambulatory care"[Title/Abstract]) | AND |
|  | patient care | ("Patient Care"[MeSH Terms] OR "clinical encounter"[Title/Abstract] OR "patient encounter"[Title/Abstract] OR "healthcare improvement"[Title/Abstract] OR "patient care management"[Title/Abstract] OR "clinical decision making"[Title/Abstract]) | AND |
| Scopus | Artificial Intelligence/ machine learning | ( TITLE-ABS-KEY ( "Artificial Intelligence" ) OR TITLE-ABS-KEY ( "AI" ) OR TITLE-ABS-KEY ( "machine learning" ) OR TITLE-ABS-KEY ( "deep learning" ) OR TITLE-ABS-KEY ( "neural networks" ) ) | AND |
|  | primary/ ambulatory care | ( TITLE-ABS-KEY ( "Outpatient Clinics, Hospital" ) OR TITLE-ABS-KEY ( "outpatient" ) OR TITLE-ABS-KEY ( "outpatient medicine" ) OR TITLE-ABS-KEY ( "primary care" ) OR TITLE-ABS-KEY ( "family medicine" ) OR TITLE-ABS-KEY ( "ambulatory care" ) ) | AND |
|  | patient care | ( TITLE-ABS-KEY ( "Patient Care" ) OR TITLE-ABS-KEY ( "clinical encounter" ) OR TITLE-ABS-KEY ( "patient encounter" ) OR TITLE-ABS-KEY ( "healthcare improvement" ) OR TITLE-ABS-KEY ( "patient care management" ) OR TITLE-ABS-KEY ( "clinical decision making" ) ) | AND |
| CINAHL | Artificial Intelligence/ machine learning | (TX "Artificial Intelligence" OR TX "AI" OR TX "machine learning" OR TX "deep learning" OR TX "neural networks") | AND |
|  | primary/ ambulatory care | (TX "Outpatient Clinics, Hospital" OR TX "outpatient" OR TX "outpatient medicine" OR TX "primary care" OR TX "family medicine" OR TX "ambulatory care") | AND |
|  | patient care | (TX "Patient Care" OR TX "clinical encounter" OR TX "patient encounter" OR TX "healthcare improvement" OR TX "patient care management" OR TX "clinical decision making") | AND |
| ClinicalTrials.gov | Artificial Intelligence/ machine learning | ("Artificial Intelligence" OR "AI" OR "machine learning" OR "deep learning" OR "neural networks") | AND |
|  | primary/ ambulatory care | (“Outpatient Clinics, Hospital" OR "outpatient" OR "outpatient medicine" OR "primary care" OR "family medicine" OR "ambulatory care") | AND |
|  | patient care | (“"Patient Care" OR "clinical encounter" OR "patient encounter" OR "healthcare improvement" OR "patient care management" OR "clinical decision making") | AND |

*Supplemental Table B: Inclusion and exclusion criteria*

| **Category** | **Inclusion Criteria** | **Exclusion Criteria** |
| --- | --- | --- |
| Document Type | Peer-reviewed publications  Primary studies  Research articles | Physician letters or notes  Book or book chapter  Reviews (ex: systematic reviews, scoping reviews, etc.)  Surveys |
| Population | Ambulatory setting  Outpatient setting  Primary care  Mental health care in ambulatory setting  Rehabilitative care in ambulatory setting  EHR analysis within ambulatory clinical care (using EHR with AI) | Inpatient setting  Emergency department  Nursing care facilities  End of life care  Palliative care  Specialty care or condition (ex: COPD, neurology, etc.)  EHR analysis external to ambulatory care settings  Focus groups |
| Subject Area | Medicine  Clinical Informatics/ Engineering  Neuroscience  Psychology  Dentistry | Non-healthcare related fields (ex: environmental sciences, agricultural sciences, social sciences, etc.)  Nursing  Pharmacology & Pharmaceutics  Veterinary  Health Education  Healthcare management, finance or policies |
| Intervention/Exposure | Artificial intelligence  Deep learning  Machine learning  Neural networks |  |
| Outcome | Patient care enhancement/ satisfaction  Provider enhancement/ satisfaction | Patient hospitalization (context specific)  Performance assessment of healthcare personnel or programs  Provider/ Patient perspectives or interviews |

*Supplemental Table C: Characteristics of Included Publications*

| Attribute | References |
| --- | --- |
| Publication year | 2019, 2020, 2021, 2022, 2023-2024 |
| Study location | United states, Europe, Asia, Canada |
| Visit work | History review, symptom assessment, diagnosing, predicting, testing, treatment, education, follow-up planning, documentation |
| Technology used | AI-Based Models*, Machine Learning (ML) Models, Deep Learning (DL) Models, Natural Language Processing (NLP), Data and Sensor Technology, Mobile Health (mHealth), Clinical Decision Support Systems (CDSS), Wearable Devices, Predictive Models, Reinforcement Learning, Hybrid Neural Architecture, Generative AI  *AI-based models, Machine Learning (ML) Models, and Deep Learning (DL) Models are often used together in the same study, leading to multiple counts for a single study. Similarly, Data and Sensor Technology might be combined with Wearable Devices or Clinical Decision-Support Systems (CDSS), contributing to overlapping categories. |
| Study design | Quantitative (n=40), Mixed Methods (n=6), Qualitative (n=1), Protocol (n=1), Review (n=1), Case Study (n=1), Systematic Review (n=2) |
| Innovation | Decision algorithm development, Multicenter validation, Hybrid neural architecture, ECG-based machine learning, AI influence analysis, Natural language processing, Big data analytics, AI implementation in care, Deep learning support, mHealth and AI, Deep learning assistance, Machine learning models, Model validation, AI-based education, EHR data analysis, Study population enrichment, Wearable devices integration, Model application development, AI-driven toolkits, ChatGPT comparison, Screening tool implementation, Clinical trial, Tool validation, Risk prediction model, Performance comparison, Data-driven models, Prediction models, Deep learning validation, Multi-method validation, Symptom extraction, AI counselor, Nonrandomized trial, Diagnostic accuracy, Prediction model validation, Machine learning treatment, Clinical notes approach, Recurrent neural network, Reinforcement learning, Screening pilot, Multivariable models, image-based diagnosis |

Supplemental Figure A Sources of Included Articles
